# HIV Surveillance Among Pregnant Women Attending Antenatal Clinics: Evolution and Current Direction

**DOI:** 10.2196/publichealth.8000

**Published:** 2017-12-05

**Authors:** Jacob Dee, Jesus M Garcia Calleja, Kimberly Marsh, Irum Zaidi, Christopher Murrill, Mahesh Swaminathan

**Affiliations:** ^1^ Division of Global HIV and TB US Centers for Disease Control and Prevention Atlanta, GA United States; ^2^ HIV Strategic Information and Planning World Health Organization Geneva Switzerland; ^3^ Strategic Information and Monitoring Division Joint United Nations Programme on HIV/AIDS Geneva Switzerland; ^4^ Office of the US Global AIDS Coordinator Washington, DC United States

**Keywords:** HIV, surveillance, prenatal, pregnant women, ethics

## Abstract

Since the late 1980s, human immunodeficiency virus (HIV) sentinel serosurveillance among pregnant women attending select antenatal clinics (ANCs) based on unlinked anonymous testing (UAT) has provided invaluable information for tracking HIV prevalence and trends and informing global and national HIV models in most countries with generalized HIV epidemics. However, increased coverage of HIV testing, prevention of mother-to-child transmission (PMTCT), and antiretroviral therapy has heightened ethical concerns about UAT. PMTCT programs now routinely collect demographic and HIV testing information from the same pregnant women as serosurveillance and therefore present an alternative to UAT-based ANC serosurveillance. This paper reports on the evolution and current direction of the global approach to HIV surveillance among pregnant women attending ANCs, including the transition away from traditional UAT-based serosurveillance and toward new guidance from the World Health Organization and the Joint United Nations Programme on HIV/AIDS on the implementation of surveillance among pregnant women attending ANCs based on routine PMTCT program data.

## Introduction

Countries affected by the human immunodeficiency virus (HIV) epidemic require information on trends in HIV prevalence to monitor the course of their HIV epidemics, allocate HIV control resources, and plan programs for HIV prevention and control [1]. Over the last 20 years, HIV serosurveillance has provided valuable information about trends in HIV prevalence among pregnant women attending regular antenatal clinics (ANCs), provided key inputs to HIV epidemic modeling, and—combined with household surveys, key population surveys, and surveillance of sexually transmitted infections (STIs)—allowed programs to address key aspects of the “know your epidemic” approach of second-generation surveillance of HIV [2].

As HIV control programs expanded in coverage and quality, routine HIV testing with return of results was increasingly available to pregnant women attending ANCs. This evolution raised questions about the appropriateness of the serosurveillance approach. From an ethical perspective, testing pregnant women for HIV without their consent or the return of results became increasingly untenable. From a sustainability perspective, the cost and effort of serosurveillance were called into question, given that the provision of HIV testing and the registering of test results were being accomplished routinely.

This paper attempts to summarize the evolution of the global approach to HIV surveillance among pregnant women attending ANCs, including origins of ANC serosurveillance, program expansion and questioning of the serosurveillance approach, and movement to consensus, culminating in the 2015 World Health Organization (WHO) guidelines recommending the use of routine program data for HIV surveillance among pregnant women.

## The Evolution of HIV Sentinel Surveillance Among Pregnant Women Attending Antenatal Clinics

In 1988, 2 years after the global program on acquired immunodeficiency syndrome (AIDS) was founded by the WHO, epidemiologists recommended the establishment of HIV sentinel surveillance systems among different health services to monitor the HIV epidemic in select populations, including pregnant women [3]. This strategy identified the availability of remnant blood from routine syphilis testing among pregnant women attending ANCs as an opportunity to conduct HIV surveillance. ANC attendees were thought to represent an accessible cross section of healthy, sexually active women in the general population, and results from ANC HIV sentinel serosurveillance were considered a general proxy for HIV prevalence in the underlying community [4,5]. The names and personally identifying information of the pregnant women were removed in such a way that it would not be possible to link a specimen to a specific person, thus assuring the confidentiality of the pregnant women. This strategy came to be known as unlinked anonymous testing (UAT) [6].

International guidelines for HIV sentinel serosurveillance describing UAT were disseminated by WHO in 1989 and updated in 2003 [2,7]. These guidelines presented UAT as an important and effective method of public health surveillance that, if approved by national ethics committees and implemented in accordance with rigorous standards, did not compromise core bioethical principles. Foremost among these standards was the permanent delinking of personally identifying information from surveillance data and the safeguarding of the confidentiality and privacy of pregnant women sampled by surveillance. UAT-based ANC serosurveillance was a key element of HIV surveillance for at least 20 years, particularly in countries with generalized epidemics where serosurveillance was conducted on an annual or biannual basis.

UAT-based ANC serosurveillance has provided valuable information about trends in HIV prevalence among pregnant women attending ANCs and served as a proxy for trends in prevalence among the general population, enabling countries to monitor the course of their HIV epidemics; advocate for support for HIV programming and resources; allocate resources for design; and measure the effectiveness of HIV control and prevention interventions [6,8]. Between 1993 and 2012, at least 84 ANC serosurveillance activities were conducted in sub-Saharan Africa [9]. Indeed, before the scale-up and destigmatization of routine HIV screening at ANC in sub-Saharan Africa, the UAT approach was important in limiting participation biases in serosurveillance HIV estimates. ANC serosurveillance has also been used as a key data source for the Joint United Nations Programme on HIV/AIDS (UNAIDS) and individual countries to model national, regional, and global HIV incidence and trends, using the UNAIDS-supported Spectrum modeling software (Avenir Health, Glastonbury, CT) [10-13].

## Concerns About UAT-Based ANC Serosurveillance

Despite the historical contributions of UAT-based ANC serosurveillance to monitor the HIV epidemic, its use as a surveillance strategy has been questioned on ethical grounds since the early 1990s [14]. Critics viewed this strategy as inconsistent with the core bioethical principle of respect for persons (as outlined in the 1979 US Department of Health, Education, and Welfare’s *Belmont Report*, the 1991 Council for International Organizations of Medical Sciences and WHO *International Guidelines for Ethical Review of Epidemiological Studies*, and the 2009 *International Ethical Guidelines on Epidemiological Studies*), as it failed to respect the autonomy of pregnant women to make informed choices about participation in surveillance and surveillance-related testing [14-18]. The advent of effective drug therapy to prevent mother-to-child transmission of HIV in the mid-1990s increased ethical concerns about UAT as it did not provide notification of HIV test results or the opportunity for intervention [19]. Consequently, UAT-based serosurveillance among pregnant women in the United States was discontinued in 1995 [20]. However, many other countries, including high-income countries such as the United Kingdom, retained UAT and have continued to use this strategy to monitor HIV epidemics [21,22].

Over the last 15 years (spurred substantially by the creation of the Global Fund to Fight AIDS, Tuberculosis and Malaria in 2002, and the President’s Emergency Plan for AIDS Relief [PEPFAR] in 2003), coverage of HIV services has increased significantly in low- and middle-income countries, including HIV testing among women attending ANCs, prevention of mother-to-child transmission (PMTCT), and antiretroviral therapy (ART). The introduction of provider-initiated HIV testing and counseling within ANC services also contributed to substantial increases in coverage of HIV testing among pregnant women attending ANCs. In 2015, an estimated 16,738,964 pregnant women in sub-Saharan Africa had an HIV test and received their results at their first ANC visit [23].

In 2013, WHO published Consolidated Guidelines on General HIV Care and the Use of Antiretroviral Drugs for Treating and Preventing HIV Infection, recommending immediate initiation of life-long ART (option B+) for all pregnant women diagnosed with HIV [24]. As of April 2017, all priority countries have adopted such policies [25]. More recently, in 2015, WHO recommended initiation of treatment for all people living with HIV (PLHIV) [26]. In the following year, an estimated 1,024,480 pregnant women living with HIV in sub-Saharan Africa received ARTs to prevent mother-to-child transmission of HIV [23].

The move to offer treatment for all sharpened ethical questions surrounding UAT, namely, (1) UAT did not obtain informed consent from pregnant women tested for HIV for surveillance purposes, provide them with their surveillance HIV test results, or refer women with HIV-positive surveillance test results to available HIV care, treatment, and prevention interventions and (2) information needed for surveillance (such as sociodemographic, syphilis, and HIV testing data) was largely present in routine PMTCT program records, rendering UAT-based serosurveillance redundant [18,22]. In addition, a published manuscript conducting statistical testing of differences between UAT- and PMTCT-based HIV prevalence estimates at the site level found few statistical differences [27].

In February 2009, UNAIDS and WHO held a partner consultation in Geneva, Switzerland, to review ethical issues associated with HIV testing in the context of national population surveys and sentinel surveillance among pregnant women. This meeting recommended that WHO and UNAIDS commission formal guidance on ethical issues in HIV surveillance [28]. The resulting 2013 publication, *Guiding principles on ethical issues in HIV surveillance*, states that UAT should be used for surveillance only when data from clinical settings and other studies cannot provide the necessary information and that surveillance programs implementing UAT must demonstrate that program data are not adequate for the purpose of public health surveillance [29]. In addition, WHO’s 2015 *Consolidated guidelines on HIV testing services* recommended that “HIV surveillance systems should work toward assuring that all participants in biological surveillance receive their HIV status” and specifically recommended that countries move from traditional ANC surveillance to using routine program data for surveillance among pregnant women attending ANCs [30]. Finally, in 2016, PEPFAR released a policy statement, *HIV testing in PEPFAR-supported survey and surveillance activities*, specifying that “All PEPFAR-supported survey and surveillance activities will provide participants the opportunity to receive final HIV status information generated by the activity.”

## Transition to Using Routine Program Data for Surveillance

Over the past several years, a shared vision has emerged in the global HIV surveillance community that recognizes the advantages and desirability of transitioning to using routine program data for ANC surveillance. The urgency of this transition has been underlined by the dramatic expansion in the coverage of HIV services in ANC settings.

ANC surveillance based on routine program data offers multiple potential advantages over UAT-based serosurveillance. First, this approach adheres to the WHO and UNAIDS guidelines by guaranteeing that all HIV serostatus data used for surveillance come from HIV testing that ensures—as per routine clinical practice—that pregnant women have the informed and free choice to accept or decline (ie, opt out of) testing, receive pre- and posttest counseling, receive their HIV test result, and are referred to HIV services if the test result is positive. Second, because ANC surveillance based on routine data would eliminate or substantially reduce the need for additional training, human resources, logistics, and HIV testing associated with traditional ANC serosurveillance, such a system can significantly reduce the workload and financial costs associated with monitoring the HIV epidemic. This can improve the sustainability of surveillance systems by integrating surveillance into routine activities and data systems. Third, increased use of program data for surveillance can contribute to the monitoring, use, and strengthening of routine data and routine HIV testing to benefit program implementation, program monitoring, and surveillance.

In the context of increasing global consensus on the benefits of using routine program data for surveillance, in 2013 the WHO and UNAIDS HIV Global Surveillance Working Group, in collaboration with the US Centers for Disease Control and Prevention (CDC), published guidelines for countries to assess how routine PMTCT program data can be used for surveillance. The guidelines stressed that collaboration between surveillance, monitoring and evaluation, and program (Maternal and child health [MCH], lab, PMTCT) is foundational to the use of routine data for surveillance. Cooperation among these entities is crucial to monitor and strengthen the quality and completeness of routine PMTCT program data, the accuracy of routine PMTCT HIV testing, and quality assurance (QA) for routine PMTCT HIV testing [31].

In the context of the new WHO guidance, a broad contingent of countries has conducted studies examining how to use routine program data for surveillance. Since 2013, at least 22 countries examined or are currently examining how to use PMTCT program data for surveillance, including Angola, Botswana, Burkina Faso, Cameroon, Cote D’Ivoire, Democratic Republic of the Congo, Ethiopia, Ghana, Haiti, India, Kenya, Lesotho, Mozambique, Namibia, Nigeria, Rwanda, Senegal, Sierra Leone, South Africa, Swaziland, Zambia, and Zimbabwe [27,32-39]. As of this writing, Rwanda, Botswana, and Zimbabwe have implemented, or are implementing, rounds of surveillance among pregnant women based on routine PMTCT program data.

## New WHO Guidelines on ANC Surveillance Based on Routine Program Data

In September 2013, the WHO and CDC organized a consultation of African countries that had conducted assessments of how to use routine program data for ANC surveillance. The consensus was that countries are moving toward using routine data for surveillance in the coming years, and countries in attendance requested that the WHO provide technical guidance on how to operationalize the transition and to ensure the continued quality of HIV surveillance estimates and epidemic modeling estimates such as those produced by Spectrum. To this end, *Guidelines for conducting HIV surveillance among pregnant women attending antenatal clinics based on routine programme data* was developed by WHO and UNAIDS in collaboration with other international stakeholders and ministries of health and published in 2015 [40].

This WHO and UNAIDS guidance provides comprehensive direction for surveillance programs through each step of designing and implementing ANC surveillance based on routine program data, including ethical considerations, sample size calculations, data collection, data management, QA of surveillance activities, data analysis, surveillance monitoring, and results’ interpretation and dissemination.

Importantly, the WHO and UNAIDS guidance includes the use of routine syphilis testing data for the surveillance of syphilis among pregnant women attending ANCs. Routine syphilis testing in pregnancy is considered an essential antenatal care intervention by WHO; testing for reactive syphilis serology using rapid diagnostic tests and providing treatment of seropositive women are highly effective and cost-efficient. Historically, HIV serosurveillance of ANC attendees frequently included surveillance for maternal syphilis. This integrated approach to surveillance is both practical and epidemiologically appropriate and is reinforced in the new guidance. Furthermore, in the same way that reliance on routine testing and data for HIV surveillance can provide information to improve routine services, surveillance of maternal syphilis based on routine data can help identify gaps, and drive improvements, in the provision of routine syphilis testing and treatment services.

Important elements of the new WHO and UNAIDS guidance, which include principles of ANC surveillance, operational design approaches, monitoring and QA, and ethical issues, are discussed below:

### Principles of ANC Surveillance

New WHO and UNAIDS guidance stresses three principles of ANC surveillance. First, epidemiologic information provided by ANC surveillance, combined with other sources of epidemiological data, allows surveillance programs to address key aspects of the “know your epidemic” approach of second-generation surveillance of HIV and provides a key input for modeling national and global HIV incidence and burden [41]. Second, ANC surveillance should (1) be methodologically sound to produce reliable data, (2) adhere to ethical standards, and (3) be resource-economical. Finally, ANC surveillance should be a collaborative endeavor that profoundly engages and involves program partners to strengthen routine HIV testing and routine data collection to benefit service delivery, program monitoring, and surveillance.

### Operational Design Approaches to ANC Surveillance

New WHO and UNAIDS guidance describes two methodological approaches to ANC surveillance. The first approach involves a census of ANC—the gathering of individual-level or aggregate data from all pregnant women attending all (or nearly all) ANC sites providing PMTCT services by leveraging existing above-site (eg, regional and national) data repositories such as health management information systems (HMIS) or routine reporting systems. Census is the preferred design because it is highly resource-efficient and provides complete geographical coverage of surveillance of pregnant women attending ANCs. However, census requires the availability of routine PMTCT HIV testing and high-quality routine data at all (or nearly all) ANC sites. The guidelines encourage countries to strengthen and expand routine testing and data systems to progress, over time, toward a census approach.

A census based on individual-level data is preferable because it allows subgroup analyses that take advantage of demographic parameters (eg, examining prevalence trends in young or primigravid women) and clinical variables (eg, examining the coincidence of HIV infection and syphilis antibody positivity). The complete geographic coverage of individual-level census data could be a valuable input into global efforts to create geospatial models that estimate trends in new HIV infections at the local level. Individual-level data, as compared with aggregate counts, also enable closer monitoring of surveillance data, facilitating the identification of gaps in routine data quality. However, a census based on individual-level data is only feasible in the presence of electronic medical records (EMR) systems (with a verified high degree of accuracy, completeness, and integrity) to capture individual-level ANC data and the capacity to safeguard the confidentiality of, and manage, large volumes of individual-level data. Furthermore, the coverage of EMR systems is currently limited in ANC settings. Therefore, a more feasible approach to census in the short term is to use aggregate service counts reported by an HMIS or routine reporting system that has a verified high degree of accuracy, completeness, and integrity. Aggregate data systems for program monitoring are already in place, and these data have the advantages of simplicity, economy, and the absence of personally identifying information. However, aggregate data may limit the ability of surveillance to conduct subanalyses (as aggregate data may not be structured in such a way as to permit analysis by finer age groups, parity or gravidity, or HIV and STI coinfection) or to monitor data quality.

The second design option for ANC surveillance is a sentinel surveillance design—a convenience sample of ANC sites that are chosen to represent geographical areas or populations of interest for HIV surveillance. Sentinel surveillance has the advantages of familiarity, collecting rich individual-level data, and the ability to prescreen sentinel sites (or provide additional support) to ensure readiness to participate in surveillance. In addition, sentinel surveillance can involve either retrospective or real-time data collection, which can allow surveillance programs to provide extra support to routine PMTCT activities to ensure the quality of routine PMTCT testing and data that are used for surveillance. However, sentinel surveillance requires the safeguarding of personally identifying information present in individual-level data, necessitates fieldwork to collect routine data from ANC sites, and does not provide representative estimates of HIV prevalence among ANC attendees at national or subnational levels. A sentinel surveillance design could be an appropriate option for countries initially transitioning to using routine program data for surveillance.

### Monitoring and Quality Assurance

Just as UAT serosurveillance included robust surveillance monitoring and QA, ANC surveillance based on routine data requires similar mechanisms to ensure the reliability of surveillance methods and estimates. Because ANC surveillance based on routine program data relies on data and activities owned and managed by nonsurveillance programs (eg, maternal and child health and PMTCT programs and the national reference laboratory), monitoring HIV surveillance among pregnant women attending ANCs is best viewed as a collaborative activity that engages these partners. Results of surveillance monitoring provide valuable information to strengthen routine activities. Surveillance monitoring can augment, reinforce, and highlight the value of routine monitoring and QA of the following: (1) the completeness of routine site-level data and data in HMIS or routine reporting systems and the appropriateness of routine data collection and reporting tools and (2) HIV rapid testing, including accuracy of testing, adherence to appropriate testing algorithms and practices, staff training, record keeping, and stock management.

### Ethical Issues

All HIV surveillance activities, regardless of the data source, adhere to ethical principles of biomedical research and surveillance, including respect for persons, beneficence, and justice [29]. In the context of ANC surveillance based on routine data, the WHO and UNAIDS guidelines emphasize that surveillance should adhere to ethical standards by ensuring that HIV serostatus data used for surveillance come from HIV testing that includes, as per routine clinical practice, the informed and free choice to accept or decline (ie, opt out of) testing; pre- and posttest counseling; return of test results to the client; and referral to PMTCT and HIV treatment services if test results are positive. In addition, it is essential that the confidentiality of pregnant women whose routine data are collected for surveillance is protected. These protections include human, physical, and electronic measures to ensure confidentiality and data security at every stage of surveillance, including data collection, transfer, storage, analysis, and dissemination. Finally, surveillance should collect as little personally identifying information as possible, and preferably none.

## Estimating Burden of Disease

Data from ANC surveillance are a key input for modeling national, regional, and global HIV trends and burden using HIV modeling software. Continuing work is needed to evolve best methods to incorporate routine program data into HIV modeling software, calculate adjustment factors, and understand the implications of this new data source for modeled estimates [42].

## Looking Forward

The transition to ANC surveillance based on routine data is an important milestone in HIV surveillance that enhances the efficiency, integration, sustainability, and ethical approach of surveillance. This shift represents a substantial achievement in building strong routine data systems to support HIV service delivery, program monitoring, and strategic information. Routine program data also enable surveillance estimates and UNAIDS Spectrum models to provide a more geographically granular description of subnational HIV epidemics, enabling highly targeted HIV control programs.

The use of routine data for surveillance and monitoring the response to the HIV epidemic is essential to inform planning, and measure country progress, towards achieving UNAIDS 90-90-90 goals (90% of PLHIV know their HIV status, 90% of PLHIV with known status are on treatment, and 90% of PLHIV on treatment are virally suppressed) at the national and subnational levels [43]. The WHO, UNAIDS, PEPFAR, and the CDC are committed to support this process through ongoing technical collaboration.

## Abbreviations

AIDS: acquired immunodeficiency syndrome
ANC: antenatal clinic
ART: antiretroviral therapy
CDC: Centers for Disease Control and Prevention
EMR: electronic medical record
HIV: human immunodeficiency virus
HMIS: health management information system
PEPFAR: President’s Emergency Plan for AIDS Relief
PLHIV: people living with HIV
PMTCT: prevention of mother-to-child transmission
QA: quality assurance
STI: sexually transmitted infection
UAT: unlinked anonymous testing
UNAIDS: United Nations Joint Programme on HIV/AIDS
WHO: World Health Organization
